# Case of Abortion—One Fœtus, with Two Placentas

**Published:** 1840-03-21

**Authors:** William Zollickoffer


### CASE OF ABORTION—ONE FŒTUS, WITH TWO PLACENTAS.

BY WILLIAM ZOLLICKOFFER. M. D.

To the Editors of the Medical Examiner.

*Middleburg*, (*Md.*) 21st March, 1840.

GENTLEMEN:—The following very singular occurrence is communicated to you, for the purpose of insertion in the "Medical Examiner," if you deem it worthy of publicity.

Very respectfully,

Wm. Zollickoffer.

During an obstetric practice of twenty-three years, I have delivered six hundred and fifty-one women, and I never recollect to have witnessed a phenomenon analogous to the one which is the subject of this paper.

On the 17th instant, Mr. Peter Ogle called on me to visit his wife, who, he said, was "about to lose her little one, and is flooding very much." The communication of this intelligence caused me to delay as little as possible in reaching his house, about three miles distant from my residence. Fortunately for Mrs. O., the fœtus was expelled some time before my arrival, and the hæmorrhage had ceased as a consequence of the uterus having contracted to an extent sufficient to check the alarming sanguineous flood, which she was, as she said, "afraid would end her life before you could see me." Mrs. O.'s situation being such as to prevent her from having the necessary attendants, on such occasions, happily for me, was the cause of the cloths remaining until I removed them. On examining their contents, I found a very small fœtus, with all the appendages enveloped in the clotted blood. Here I discovered *two distinct* placentas. The umbilical cord, about two inches from the umbilicus, bifurcated, each ramification entering a *distinct* placenta. From the size of the fœtus, I supposed Mrs. O. had been pregnant about three months. This opinion corresponded with her calculation. I have witnessed three twin cases, each having only one placenta; but never, before the case of Mrs. O., had the pleasure of viewing two placentas to a single child.

I have regretted exceedingly, since the occurrence of this circumstance, that I had not pocketed the curious production for preservation in alcohol.

